# Long Noncoding RNA *E2F4as* Promotes Progression and Predicts Patient Prognosis in Human Ovarian Cancer

**DOI:** 10.3390/cancers12123626

**Published:** 2020-12-03

**Authors:** Sun-Ae Park, Lee Kyung Kim, Young Tae Kim, Tae-Hwe Heo, Hee Jung Kim

**Affiliations:** 1 Laboratory of Pharmacoimmunology, Integrated Research Institute of Pharmaceutical Sciences, College of Pharmacy, The Catholic University of Korea, Seoul 03722, Korea; tjsdo37@catholic.ac.kr (S.-A.P.); 1202dlruddl@catholic.ac.kr (L.K.K.); 2 Institute of Women’s Life Medical Science, Division of Gynecologic Oncology, Department of Obstetrics and Gynecology, Yonsei University College of Medicine, Seoul 03722, Korea; ytkchoi@yuhs.ac

**Keywords:** ovarian cancer (OC), long noncoding RNAs (lncRNAs), E2F4 antisense (*E2F4as*), epithelial–mesenchymal transition (EMT), biomarker

## Abstract

**Simple Summary:**

LncRNA is a promising biomarker that predicts the prognosis of a variety of cancers, but the important role of E2F4antisense lncRNA in cancer remains unclear. Therefore, we decided to explore the role of E2F4as lncRNA in the blood of an ovarian cancer patient. We found that E2F4as was highly expressed in ovarian cancer patients, and that the higher the expression of E2F4as, the worse the patient’s prognosis. In addition, we observed that downregulation of E2F4as in ovarian cancer cells reduced cell proliferation, invasion and migration, decreased expression of EMT-related genes, and increased apoptosis. These findings suggest that E2F4as may be a predictive biomarker in the blood of ovarian cancer patients, and have shown the potential to promote tumor aggression through EMT-related mechanisms.

**Abstract:**

(1) Background: LncRNAs could be a promising biomarker to predict the prognosis of various cancers. The significance of *E2F4antisense* lncRNA remains unclear in cancer. In this study, we examined the expression level of *E2F4as* in the serum of ovarian cancer patients and the functional role of *E2F4as*. (2) Methods: Serum samples were obtained from 108 OC patients and 32 normal patients to measure the expression of *E2F4as* in the serum. Ovarian cancer cells were used to investigate the role of *E2F4as* in cell proliferation, invasion, migration and apoptosis, and the expression of *E2F4as* was knocked down using RNA interference. In addition, *E2F4as* knockdown cell lines were used in in vivo experiments. (3) Results: The expression of *E2F4as* was significantly higher in the serum of OC patients than in that of control patients (*p* < 0.05). The knockdown of *E2F4as* in ovarian cancer cells led to a decrease in cell proliferation, invasion and migration and an increase in apoptosis. *E2F4as* knockdown also reduced the expression of epithelium–mesenchymal metastasis (EMT) genes. (4) Conclusion: These findings highlight the clinical significance of *E2F4as* in predicting the prognosis of OC patients and suggest its potential in promoting tumour aggressiveness by the regulation of EMT-related mechanisms.

## 1. Introduction

Ovarian cancer is globally the sixth most common cancer in women, the second most common gynaecologic malignancy in women, and the most fatal tumour of the female reproductive system [1]. Ovarian cancer is the most fatal gynaecologic malignancy due to its aggressive metastasis, recurrence, and drug resistance [2]. The five-year survival rate of below 40% applies to late stage ovarian cancer, so an effective alternative strategy is needed to overcome tumour penetration and aggressive metastasis [3]. Therefore, an early diagnosis of ovarian cancer may play an important role in improving the prognosis and survival rate of ovarian cancer patients. In this regard, predicting the risk of ovarian cancer using patient serum could be one of the easiest methods for the early diagnosis of ovarian cancer.

Long noncoding RNAs (lncRNAs) constitute a heterogeneous group of genomic transcripts longer than 200 nucleotides without a protein-coding function [4,5]. Unlike short noncoding RNAs, such as microRNAs, the functional role of lncRNAs was underestimated because they were initially considered transcriptional noise in the genome [6]. However, recent studies showed their importance not only in normal function but also in the regulation of various biological processes such as stem cell proliferation, apoptosis, cell migration, and metastasis in cancer cells. They also supported new evidence that changes in lncRNA expression occur in a variety of human cancers, and that expression patterns are associated with cancer progression and metastasis [7].

The E2F family of transcription factors is known to be involved in the regulation of the cell cycle, proliferation, differentiation, DNA repair, and apoptosis [8,9]. E2F transcription factors are key regulators of cell cycle progression that regulate gene expression required for G1/S metastasis [10]. Among the eight members of the E2F family, E2F1-3a is a transcriptional activator, while E2F3b-7 was found to inhibit downstream gene transcription [11,12,13]. E2F4 constitutes a defined subset of the family. It was demonstrated that E2F4 accumulate sequentially in the nucleus of cycling cells and controls gene expression during cell-cycle exit. Now, little is understood about individual biochemical and biological functions [14]. This study focused on studying the “antisense” strand of the E2F4 gene cluster known as the noncoding RNA gene. This study was conducted to investigate the role of E2F4 in carcinogenesis since little is known about the function of locally present lncRNAs, and no studies have been conducted on *E2F4as* (antisense). Induction of *E2F4as* by Wnt signalling may contribute to carcinogenesis by reducing levels of the E2F4 cell cycle repressor in colorectal cancer [15]. Wnt/β-catenin signalling also promotes epithelial–mesenchymal metastasis (EMT) by inducing the expression of EMT transcription factors. EMT contributes to the invasive and metastatic spread of colorectal cancer, and is associated with chemotherapy resistance [16].

In this study, we investigated the functional role of the lncRNA *E2F4as* in the progression of ovarian cancer. Our results indicated that *E2F4as* occurs in ovarian cancer probably through the EMT signalling pathway for apoptosis, cell growth, invasion, and metastasis. Our study suggests that *E2F4as* in the serum of ovarian cancer patients is a promising biomarker for early diagnosis and treatment.

## 2. Results

### 2.1. E2F4as Overexpressed in Ovarian Cancer Patient Serum

**Figure 1:**
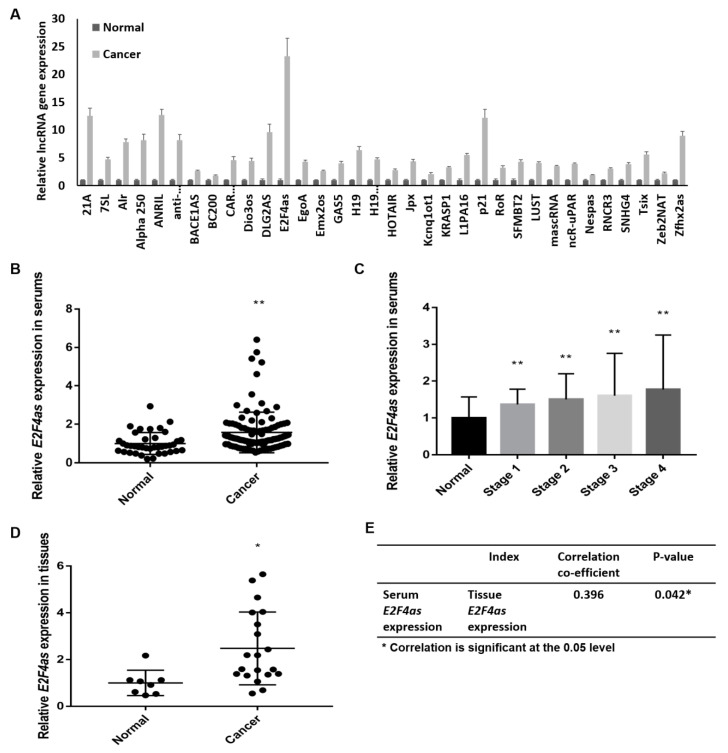
Expression of lncRNA *E2F4as* in the serum of ovarian cancer patients. (**A**) LncRNAs profile in ovarian patient samples identified by qRT-PCR in the serum of 38 ovarian cancer patients and 8 normal patients; (**B**) Expression of lncRNA *E2F4as* was measured by qRT-PCR technology in the serum of 108 ovarian cancer patients and 32 normal patients; (**C**) Expression of lncRNA *E2F4as* in the serum of ovarian cancer patients per cancer stage detected using qRT-PCR in 108 ovarian cancer patients and 32 normal patients; (**D**) Expression of lncRNA *E2F4as* was measured using qRT-PCR technology in the tissues of 20 ovarian cancer patients and 8 normal patients; * *p* < 0.05, ** *p* < 0.01 vs. normal. (**E**) Correlation between *E2F4as* gene expression in the serum and tissue clinical induce; * *p* < 0.05.

The serum of 32 patients with ovarian cancer and 8 patients with corresponding normal was used for the LncProfiler qPCR Array. We examined LncProfiler qPCR Array to profile the expression of 90 major lncRNAs with three biological replicates. Results indicated that the expression of *E2F4as* is highly upregulated in ovarian cancer patients (Figure 1A). The serum of patients with ovarian cancer was further collected, and *E2F4as* expression in the serum of ovarian cancer patients was analysed to determine if it was related to clinical pathological features. We evaluated the expression of *E2F4as* in ovarian cancer patient serum (*n* = 108) and corresponding normal patient serum (*n* = 32) using qRT-PCR on the collected patient serums. As a result, *E2F4as* in ovarian cancer patient serum was more than 1.6-fold that of normal patient serum (*p* < 0.01) (Figure 1B) In addition, the expression of *E2F4as* for each stage of ovarian cancer was confirmed. Compared to normal patients, the expression of *E2F4as* was significantly higher in the late stage in ovarian cancer patients (Figure 1C). The expression of *E2F4as* was confirmed through a comparison of tissues between normal patients and ovarian cancer patients to confirm that the tissues also showed the same pattern as that of the serum. Like serum, the expression of *E2F4as* was elevated in the tissues of ovarian cancer patients. (Figure 1D). *E2F4as* expression was confirmed in the tissues of 20 ovarian cancer patients and 8 normal patients who had the same results in the serum. A positive correlation was noted between the serum and the tissues, with a correlation coefficient of 0.0396 and a significance probability of 0.042 (*p* < 0.05) (Figure 1E).

### 2.2. E2F4as Levels Elevated in Patient Serum with Ovarian Cancer Having Poor Prognoses

Next, we examined the relationship between *E2F4as* expression and outcomes. Clinicopathological and outcome information was available for 108 ovarian cancer patients. The lengths of the follow-up periods were 1–60 months. Clinicopathological features were compared between high- and low-*E2F4as*-expression groups. Patients serum with high *E2F4as* expression exhibited higher rates of stage (*p* = 0.006) and lymph node metastasis (*p* = 0.004) relative to patient serum with low *E2F4as* expression; this relationship was statistically significant (Table 1). Therefore, these findings suggest that increased *E2F4as* levels are associated with the aggressive behaviours of ovarian cancer.

**Figure 2:**
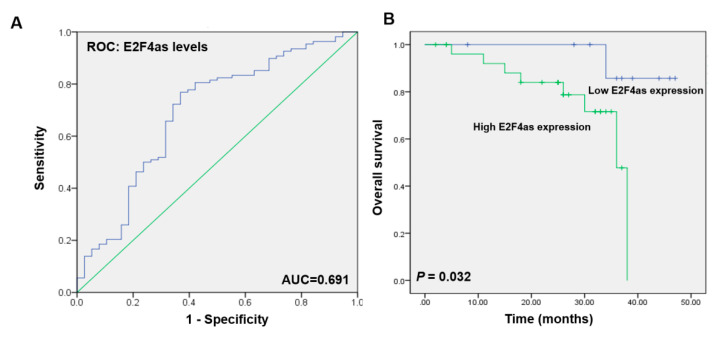
Overall survival of *E2F4as* expression in ovarian cancer patient serum. (**A**) Receiver operating characteristic (ROC) curve for prognosis prediction of patients using *E2F4as* level. Area under the curve (AUC) shown in plots. (**B**) Relative *E2F4as* expression and its clinical significance based on Kaplan–Meier overall survival curves of patients with ovarian cancer and different levels of *E2F4as*.

The risk model for *E2F4as* data showed a predicted area under curve (AUC) of 0.691 (*p* = 0.0004873; Figure 2A). Kaplan–Meier survival analysis showed that ovarian cancer patients with low *E2F4as* levels had longer overall and disease-free survival than did ovarian cancer patients with high *E2F4as* levels (*p* = 0.032; Figure 2B). In addition, univariate and multivariate analysis was performed using the Cox proportional risk model; it was shown that *E2F4as* expression significantly affects *E2F4as* expression, Stage, metastatic lymph node metastasis and menopause (*E2F4as* expression, multivariate HR = 0.218 (0.048–0.996), *p* = 0.049; Stage, multivariate HR = 2.130 (1.005–4.513), *p* = 0.048; lymph node metastasis, univariate HR = 4.527 (1.458–14.056), *p* = 0.004, multivariate HR = 4.324 (1.392–13.425), *p* = 0.011; menopause, univariate HR = 7.368 (0.957–56.723), *p* = 0.024; Table 2). These data suggest that *E2F4as* is an independent prognostic factor for overall survival for lymph node metastasis.

### 2.3. Inhibition of E2F4as Could Suppress Proliferation and Metastasis of Ovarian Cancer Cells

**Figure 3:**
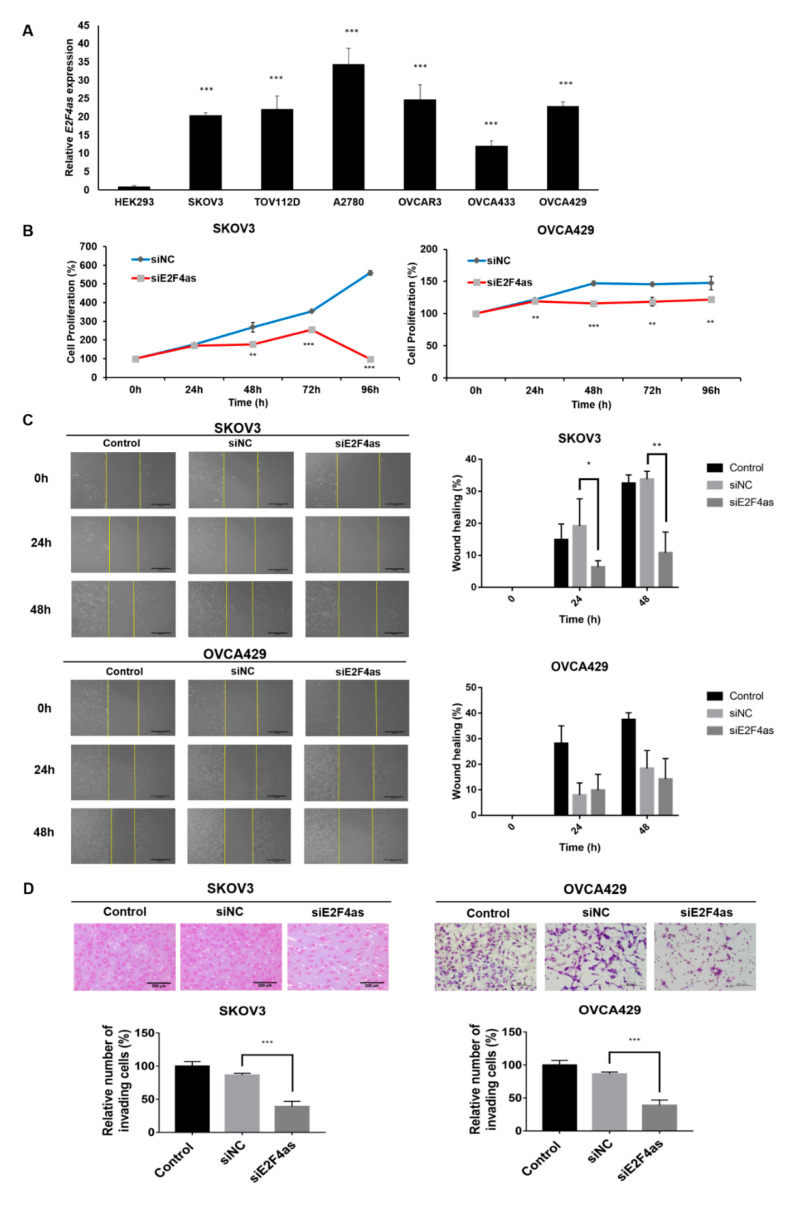
Inhibition of *E2F4as* could suppress proliferation and metastasis of ovarian cancer cells. (**A**) Expression of lncRNA *E2F4as* in six ovarian cancer cell lines and one human embryonic kidney cell line was detected by qRT-PCR. (**B**) CCK-8 assay used to detect proliferation ability of SKOV3 and OVCA429 cells. (**C**) Wound healing assay used to measure migration ability. Yellow colour line indicates width of the wound. (**D**) Cell invasion observed under optical microscope. Matrigel invasion assay used to determine invasion after 48 h of incubation of *E2F4as* knockdown SKOV3 and OVCA429 cells. Matrigel invasion assay results showing percentage of each cell line. Data expressed as mean ± standard deviation; *p < 0.05, **p < 0.01, *** *p* < 0.001 vs. siNC.

To investigate the expression of *E2F4as* in ovarian cancer cells, qRT-PCR was performed in several cell lines. SKOV3, TOV112D, A2780, OVCAR3, and OVCA429 had higher expression levels of *E2F4as* than the control (HEK293) (Figure 3A). Among the cell lines with increased expression of *E2F4as*, SKOV3 and OVCA429—which are serous-type cell lines—were selected for the experiment. siE2F4as selected siE2F4as-5, which had the most decreased expression of *E2F4as* through qRT-PCR among several siRNAs (Supplementary Materials Figure S1A–D). The proliferation of siE2F4as-transfected ovarian cancer cells and siNC was measured by CCK-8 assay. Knockdown of *E2F4as* inhibited cell proliferation in SKOV3 and OVCA429 cell lines, relative to siNC (Figure 3B). Next, the effects of *E2F4as* on the invasive and migratory behaviour of SKOV3 cancer cells were assessed by Matrigel invasion and wound healing assays. Wound healing assays showed larger width that demonstrated the decreased migration of ovarian cancer cells via *E2F4as* downregulation (Figure 3C). The invasive capacity of SKOV3 and OVCA429 cells decreased after 48 h upon the downregulation of *E2F4as* (Figure 3D).

### 2.4. Downregulated E2F4as Increases Cell Cycle Arrest and Apoptosis in Ovarian Cancer Cells

**Figure 4:**
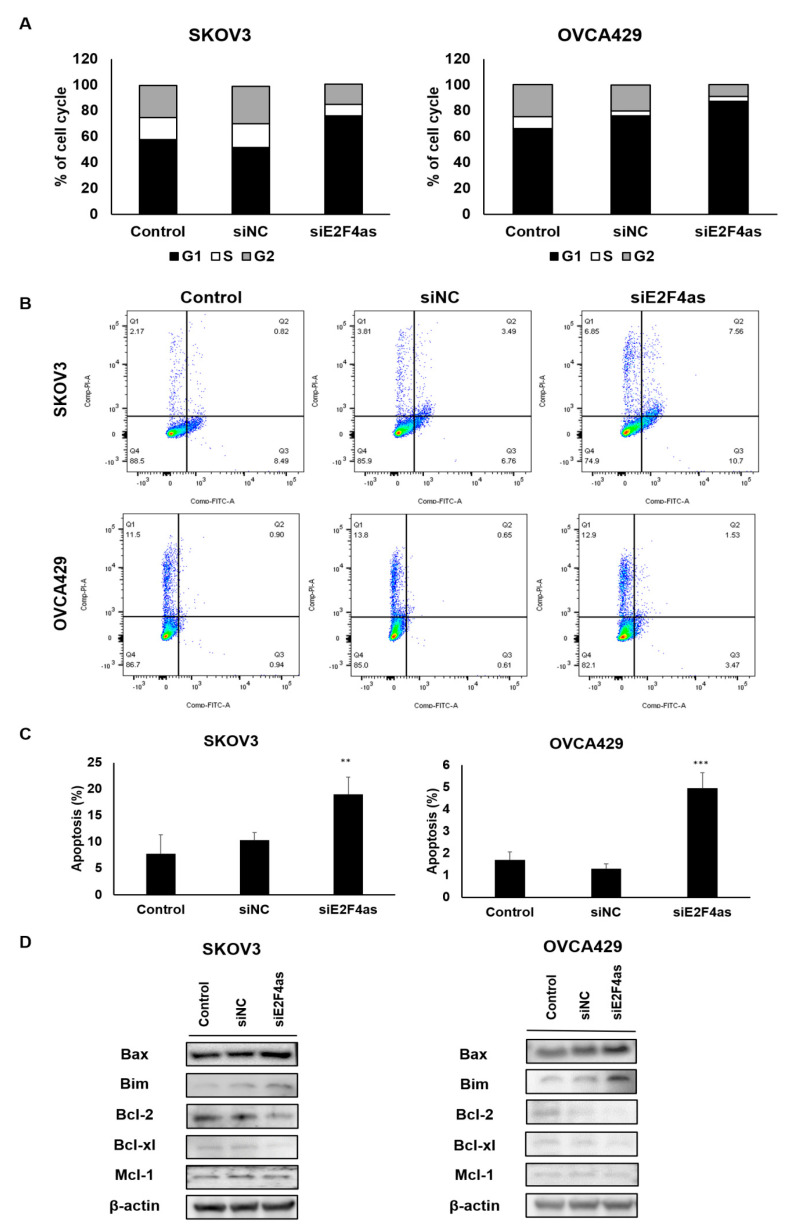
Inhibition of *E2F4as* promote apoptosis of ovarian cancer cells. (**A**) Cell cycle was arrested in the G1 phase after transfection of siE2F4as in ovarian cancer cells. (**B**) Ovarian cancer cells were transfected with siNC, siE2F4as, and control in triplicate for 48 h to detect cell apoptosis using FITC Annexin V apoptosis kit. (**C**) Levels of apoptosis in siE2F4as analysed using FACS Canto II. Data expressed as mean ± standard deviation; ** *p* < 0.01, *** *p* < 0.001 vs. control. (**D**) Protein lysates obtained from SKOV3 and OVCA429 cells 48 h after transfection with *E2F4as* siRNA (30 nM) or negative control. Levels of proteins in apoptosis-associated gene analysed using Western blotting. Data are representative of three independent experiments.

The E2F family is known to influence cell cycle and cell proliferation [10]. E2F4, one of the E2F repressors, is critical to maintain cell cycle arrest in G0/G1. We hypothesize that *E2F4as* could affect E2F4 levels and tested E2F4 mRNA levels after downregulating *E2F4as*. As a result, knockdown of *E2F4as* increased E2F4 expression in SKOV3 and OVCA429 cell lines compared to siNC (Figure S1E,F). The potential influences of *E2F4as* on cell cycle and apoptosis of ovarian cancer cell lines were elucidated. In contrast to control, the number of cells in the G0/G1 stage in siE2F4as increased significantly, whereas the cells in the S stage decreased after siE2F4as transfection, indicating a quiescent cell cycle (transfection with NC; Figure 4A). We further investigated the potential effect of *E2F4as* on ovarian cancer cell apoptosis. The results of flow cytometry analysis indicated by Annexin V-FITC/PI double staining revealed that *E2F4as* knockdown significantly induced the apoptosis of SKOV3 and OVCA429 cells compared with the control (transfection with NC; Figure 4B,C). Downregulation of *E2F4as* also notably reduced levels of Bcl-2 and Bcl-xl, and elevated levels of Bax and Bim in ovarian cancer cells (Figure 4D and Figure S2). There was no significant difference in Mcl-1. It is speculated that *E2F4as* knockdown inhibits the proliferation ability of ovarian cancer cells by arresting cell cycle and inducing apoptosis.

### 2.5. Effects of Knockdown E2F4as on EMT-Associated Genes in Ovarian Cancer Cells

**Figure 5:**
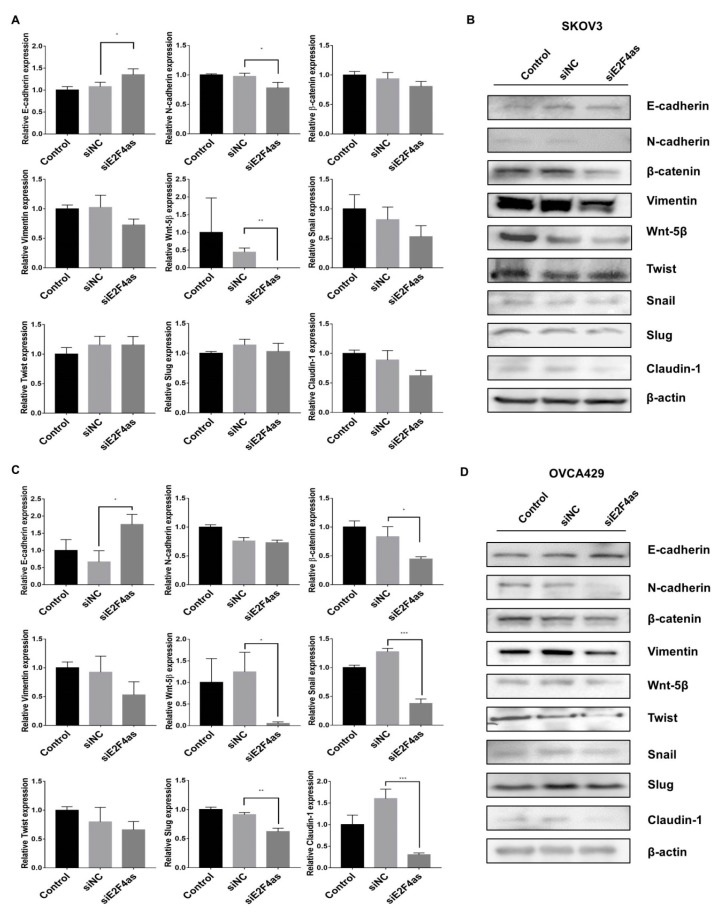
Effects of knockdown *E2F4as* on epithelial–mesenchymal metastasis (EMT)-associated genes in SKOV3 and OVCA429 cells. (**A**,**C**) Levels of E-cadherin, N-cadherin, β-catenin, Wnt-5β, Twist, Snail, Slug, Vimentin, and claudin-1 analysed by qRT-PCR in SKOV3 and OVCA429 cells 24 h after transfection with *E2F4as* siRNA (30nM) or negative control. Data expressed as mean ± standard deviation; * *p* < 0.05, ** *p* < 0.01, *** *p* < 0.001 vs. siNC. (**B**,**D**) Protein lysates obtained from SKOV3 and OVCA429 cells 48 h after transfection with *E2F4as* siRNA (30nM) or negative control. Levels of E-cadherin, N-cadherin, β-catenin, Wnt-5β, Twist, Snail, Slug, Vimentin, and claudin-1 analysed by Western blotting. Data are representative of three independent experiments.

To determine the mechanism by which *E2F4as* inhibits a malignant phenotype in ovarian cancer cells, we assessed the status of important signalling cascades controlled by EMT in siE2F4as cells. *E2F4as* knockdown in SKOV3 cells resulted in increased expression of E-cadherin; decreased expression of N-cadherin and Wnt-5β in mRNA; decreased expression of N-cadherin, β-catenin, Vimentin, Wnt-5β, Snail, and claudin-1 expression protein (Figure 5 and Figure S3). *E2F4as* knockdown in OVCA429 cells resulted in increased expression of E-cadherin; decreased expression of β-catenin, Wnt-5β, Snail, Slug, and claudin-1 in mRNA; increased expression of E-cadherin; and decreased expression of N-cadherin, Wnt-5β, Vimentin, Snail, Slug, Twist, and claudin-1 expression protein (Figure 5 and Figure S3). These results showed that *E2F4as* is related to the EMT signalling pathway. It is thought that *E2F4as* is involved in EMT, and affects cancer metastasis and migration.

### 2.6. Silencing of lncE2F4as Inhibits OVCA429 Tumour Growth In Vivo

**Figure 6:**
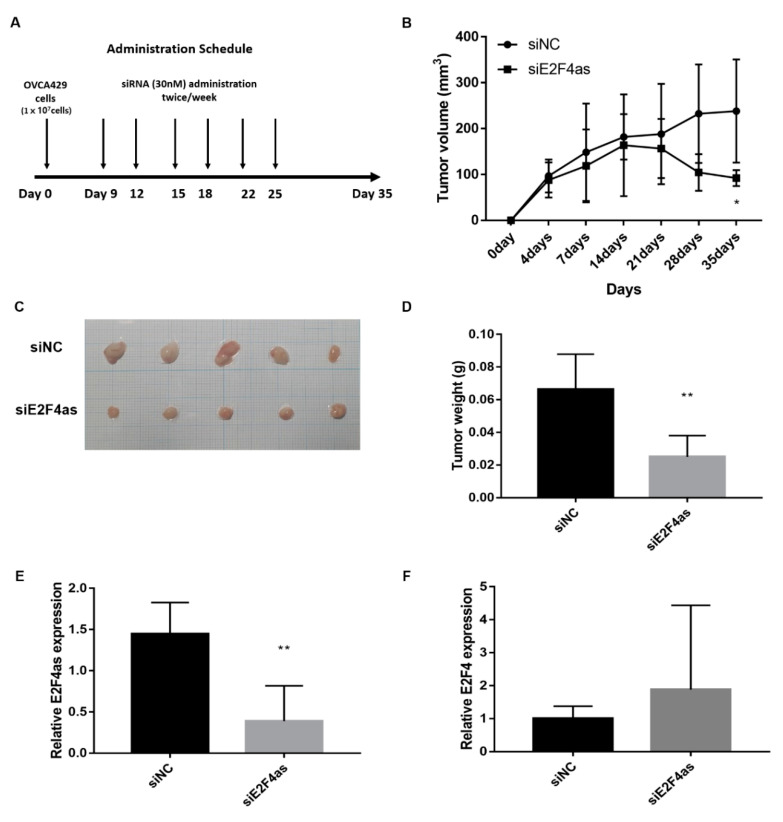
Lnc*E2F4as* inhibited OVCA429 tumour growth in vivo. (**A**) Flowchart of the experimental protocol for the xenograft model. OVCA429 cells (1 × 10^7^) were injected subcutaneously into Balb/c-nude mice with an equal volume of PBS. When tumours had formed 100 mm^3^ later, injected intratumoural siRNA. (**B**) Tumour volumes were calculated from calliper measurements. (**C**) Photograph of tumours are presented. (**D**) Tumour weight. Data are expressed as mean SD (*n* = 5). (**E**) The expression of *E2F4as* was measured by qRT-PCR in xenograft model tumour tissues * *p* < 0.05, ** *p* < 0.01 vs. siNC. (**F**) The expression of E2F4 was measured by qRT-PCR in xenograft model tumour tissues.

To determine whether *E2F4as* knockdown inhibits ovarian tumour growth in vivo, the growth of ovarian tumour xenografts in treatment siNC and siE2F4as groups was compared. Once the subcutaneous tumour had grown to a measurable level, measurement of tumour volume was started using a calliper and continued until the tumour was removed on Day 35. Then, the weight of the tumour tissue was checked. The results showed that the xenografts of the *E2F4as*–siRNA group had lower tumour volume on Day 35 than the negative xenografts did (* *p* < 0.05; Figure 6B). In addition, this showed that the average weight of tumours derived from cells of the *E2F4as*–siRNA group was significantly reduced compared to the weight of tumours derived from cells of the negative and blank controls (** *p* < 0.01; Figure 6D). The expression of *E2F4as* was confirmed by extracting mRNA from tumour tissues, and it was confirmed that the expression of *E2F4as* was significantly decreased in the siE2F4as group (** *p* < 0.01; Figure 6E). On the other hand, the level of E2F4 mRNA was increased in the siE2F4as group, but it was not statistically significant (Figure 6F). Next, we assessed the effect of *E2F4as* knockdown on the expression of EMT-related markers in xenograft mouse cancer tissues using qRT-PCR. *E2F4as* knockdown in cancer tissues resulted in an increase in the expression of E-cadherin and a decrease in the expression of Vimentin, Wnt-5β and Slug mRNA levels (Figure S4).

## 3. Discussion

Recently accumulated evidence has suggested that lncRNA may play an important role in cell biology and human disease. In gynaecological cancer, several lncRNAs were identified, including *HOTAIR, SRA, MALAT-1, H19* and *LSINCT5* [17,18,19,20]. On the basis of these data, lncRNA is emerging as an early diagnosis and treatment target. Finding effective biomarkers for early diagnosis and prognosis is important, and the early diagnosis of ovarian cancer reduces mortality. Among them, liquid biopsy is currently an effective and non-invasive method. It is urgent to identify serum biomarkers with high sensitivity and specificity [21]. In this study, high-expression lncRNAs were screened using the serum of ovarian cancer patients, among which *E2F4as* was found. However, no studies exist on the clinical prognosis importance of new lncRNA *E2F4as* in ovarian cancer. *E2F4as* expression levels in ovarian cancer were higher in blood serum, and the knockdown of *E2F4as* inhibited cell proliferation and metastasis in various ovarian cancer cell lines.

E2F4 constitutes a subset of the E2F family and the E2F family is only known to be involved in the regulation of cell cycle and apoptosis [8,10,14,22]. E2F4 transcription factors is critical to maintain cell cycle arrest in G0/G1 in conjunction with members of the retinoblastoma (RB) family. E2F4 are “repressors” that prevent uncontrolled proliferation. To date, the role of *E2F4as* is unclear with E2F4, and cell cycle and apoptosis studies have been conducted to clarify the role of *E2F4as* in ovarian cancer cell lines. This study showed that *E2F4as* expression may contribute to the development of ovarian cancer by reducing E2F4 expression, reducing the level of cell cycle inhibitors, and mediating the proliferation of ovarian cancer cells.

Questions were raised as to whether *E2F4as* promotes ovarian cancer metastasis by regulating the expression of genes encoding metastasis-related proteins. EMT was thought to be one of the manual mechanisms. EMT properties were reported to contribute to cell proliferation, invasion, migration, and metastasis in various malignant tumours [23,24]. These findings highlighted the clinical relevance of *E2F4as* in predicting the detrimental prognosis of ovarian cancer, and suggest the possibility of promoting tumour aggression through the regulation of EMT-related mechanisms [25]. To date, many noncoding RNAs (ncRNAs) were recognized to control almost all levels of gene expression and pathway activation, including the activation and inhibition of EMT processes. lncRNA *XIST* was found to promote EMT through the regulation of ZEB2 by acting as a miRNA-367/141 ceRNA in non-small cell lung cancer [26]. However, the role and mechanisms of other lncRNAs in EMT, and their effect on cell invasion and metastasis in ovarian cancer are still not well understood. Our results highlighted the predictive prognosis of ovarian cancer and the clinical relevance of *E2F4as*, and suggested the possibility of promoting tumour aggression through the regulation of EMT-related mechanisms. In vitro and in vivo observations of lncRNAs showed reduced cell growth, invasion, and migration when downregulated as in this study. Taken together, the regulatory abnormal expression of EMT-related genes appears to participate in ovarian cancer cell invasion and migration in relation to *E2F4as*.

Taken together, these results suggested that *E2F4as* may play an important role in the development of ovarian cancer. However, the relatively small clinical sample size was a limitation of this study and may have reduced the power of clinical analysis. Another limitation is that only subcutaneous xenograft models had been used to investigate cancer cell behaviour in vivo. Cancer cells implanted subcutaneously allow rapid and quantitative tumour formation, making them more suitable for use in studies involving continuous measurement of tumours. In contrast, with intraperitoneal and orthotopic xenograft models it is inherently difficult to quantitatively monitor tumour growth, but these models can reveal a more relevant tumour microenvironment.

Many researchers have persevered to detect ovarian cancer early, but details of markers are not available in literature [27]. The low sensitivity of CA125 as a serum marker to detect early ovarian cancer and monitor the patient’s clinical progression remains the biggest obstacle to improving patient outcomes [27,28]. For the first time, we demonstrated the usefulness of *E2F4as* as a less invasive prognostic marker using serum samples collected from ovarian cancer patients. The findings emphasize the clinical importance of *E2F4as* in early diagnosis and prognosis prediction using the serum of ovarian cancer patients, and suggest the potential of promoting tumour aggression by the regulation of EMT-related mechanisms. This potentially yields a less invasive and inexpensive diagnostic test that can be used in combination with other outcome factors to improve patient outcomes and provide optimal treatment. Therefore, lncRNA *E2F4as* was related to the abnormal proliferation and migration of ovarian cancer, indicating that lncRNA *E2F4as* might be a promising target in treating ovarian cancer.

## 4. Materials and Methods

### 4.1. Patient Tissue and Serum Samples

All experiments were performed with the approval of the review board for human research of Yonsei University Hospital. Samples were collected between September 2012 and December 2017 from 108 ovarian cancer patients who had undergone surgery, and a control group of 32 patients with a benign gynaecologic disease. Patients with gynaecological or other primary malignant tumours and patients receiving systemic adjuvant chemotherapy were excluded from the study. Ovarian serum samples and tumour tissues were collected during gynaecological surgery. Using liquid nitrogen, patient tumour samples were snap-frozen immediately after surgery and maintained at −80 °C until RNA extraction was performed. Progression-free survival (PFS) was defined as the interval between surgical and progression data identified by imaging studies. Overall survival (OS) was defined as surgical data up to death data.

### 4.2. Ethics Approval and Consent to Participate

This study was approved by the Institutional Review Board (IBR) of Severance Hospital, Yonsei University College of Medicine (ethic code: 4-2012-0363). The need for informed consent was waived because of the low risk designated by the IRB.

### 4.3. Serum RNA Extraction and LncRNA Expression Profiling

The number of patients included for the lncRNA expression profiling was approximately 40 and 8 per group. Patient blood was placed in an RNase-free centrifuge tube, and serum was separated through centrifugation and stored at −80 °C. Serum was extracted with an RNA extraction kit (Life Technologies, Carlsbad, CA, USA). Approximately 5 mL of the serum was obtained per patient, and approximately 0.5 μg of RNA was extracted from the separated serum. The extracted RNA was examined on the expression of 90 lncRNAs using the LncRNA Profiler^TM^ qPCR Array Kit (Human) (System Biosciences, Mountain View, CA, USA). Ninety lncRNAs and 3 housekeeping genes were included and were normalized to 3 housekeeping genes. LncRNA expression profiling and data analysis were performed according to the manufacturer’s instructions.

### 4.4. Quantitative Real-Time PCR Analysis (qRT-PCR)

Serum was extracted with an RNA extraction kit (Life Technologies, Carlsbad, California, USA). Approximately 5 mL of the serum was obtained per patient, and approximately 0.5 μg of RNA was extracted from the separated serum. Also, RNA was extracted from ovarian cancer patient tissues and ovarian cancer cell lines (TRIzol^®^ reagent, Invitrogen, Waltham, MA, USA). For the reverse transcription of 0.5 μg of RNA in the serum, 1 μg of total RNA in the cell lines and 2 μg of total RNA in the tissues into first-strand cDNA, a reverse-transcription kit (Invitrogen, MA, USA) was used according to the manufacturer’s instructions; qRT-PCR was performed using SYBR^®^ Green Real-Time PCR kit (Mbiotech, Seoul, Korea) in a 20 μL reaction volume on a Bio-Red Real-Time PCR system (BioRed Laboratories, Inc., Hercules, CA, USA). The 2−∆∆CT method was used to analyse relative gene expression. It was normalized to 3 housekeeping genes (U6, GAPDH, and 18 s). Primers used for real-time PCR reactions are listed in Table S1.

### 4.5. Cell Lines

We were purchased the SKOV3 human epithelial ovarian cancer cell line from the Korean Cell Line Bank (KCLB, Seoul, Korea), and the OVCA429 and OVCA433 cell line were provided by the Korea Gynecologic Cancer Bank through the Bio and Medical Technology Development Program of the Ministry of Science, Information and Communication Technology, and Future Planning (MSIP, Seoul, Korea). The HEK293, TOV112D and OVCAR3 cell lines were purchased from the American Type Culture Collection (ATCC, Manassas, VA, USA), and the A2780 cell line was procured from the European Collection of Cell Cultures (ECACC, London, UK). SKOV3, TOV112D and A2780 cells were cultured in RPMI-1640 medium (Welgene, Seoul, Korea), and HEK293, OVCAR3, OVCA429 and OVCA433 cells were cultured in Dulbecco’s Modified Eagle’s Medium (Welgene, Seoul, Korea). All culture media were supplemented with 10% foetal bovine serum and 1% penicillin/streptomycin, and cell lines were maintained at 37 °C in a humidified atmosphere of 5% CO_2_ and 95% air. Culture medium was replaced with fresh medium every 2–3 days, and cells were used between Passages 5 and 10. OVCA429 and OVCA433 cells were used between passages 10 and 20. The subtypes of the epithelial ovarian cancer cell lines are as follows: SKOV3, serous; TOV112D, endometrioid; A2780, non-specified; OVCAR3, carcinoma; OVCA429, serous; and OVCA433, serous [29].

### 4.6. Small Interfering RNA (siRNA) Transfection

*E2F4* antisense–targeting siRNA (siE2F4as) and negative control siRNA (siNC) were obtained from Genolution (Genolution Pharmaceuticals Inc., Seoul, Korea). Cells (5 × 10^4^ cells/well) were allocated into 6-well plates and transfected with 30 nM siRNA in phosphate-buffered saline (PBS) using the G-Fectin Kit (Genolution Pharmaceuticals Inc., Seoul, Korea) in accordance with the manufacturer’s protocol. The siRNA-transfected cells were used in the in vitro assays 24 and 48 h after transfection. Experiments were repeated at least three times. The target sequence of siE2F4as is shown in Table S2.

### 4.7. Cell Proliferation Assay

A Cell Counting Kit-8 (CCK-8) assay (Dojindo Laboratories, Kumamoto, Japan) was used to evaluate cell proliferation. Cells (1000 cells/well) were assigned to 96-well plates in 100 μL complete medium. Cells were incubated for 24 h to stabilize the cells, and 48 h treatment with siRNA was followed by cell proliferation evaluation for 0, 24, 48, 72, and 96 h. An aliquot of 10 μL of CCK-8 solution was added to each well and incubated for 2 h. Absorbance was measured at 450 nm to calculate the number of viable cells in each well. The assay was performed in triplicate.

### 4.8. Wound Healing Assay and Matrigel Invasion Assay

Matrigel invasion assay was performed using BD Biocoat Matrigel Invasion Chamber (BD Bioscience, Bedford, MA, USA) according to the manufacturer’s instructions. Cells (5 × 10^4^ cells/well) were allocated into 24-well chambers in 300 μL serum-free medium. After 48 h of incubation, cells that had invaded through invasion chamber were stained using a Differential Quik Stain kit (Diff Quik, Sysmex, Kobe, Japan).

Cell migration was assessed using monolayer wound healing assay. Grow cells to 90% confluence. Incubate the cells in 10ug/mL mitomycin C (Merck Millipore, Darmstadt, Germany) prepared in complete medium for 2 to 3 h in the incubator. Wash the cells once with PBS, an artificial wound was created by scratching to the serum-free medium and constantly scratching the monolayer. Images of migrating cells were captured at 0, 24, and 48 h using a microscope.

### 4.9. Western Blot Analysis

A Radioimmunoprecipitation assay (RIPA) buffer (Thermo Fisher Scientific Inc, Waltham, MA, USA) was used to extract proteins. We measured the protein concentrations using a Pierce BCA Protein assay kit (Thermo Fisher Scientific, Waltham, MA, USA). Proteins were boiled with a 5× sample buffer, and subsequently resolved on 10% SDS-polyacrylamide gels and electrophoretically transferred to polyvinylidene difluoride membranes (Millipore, Billerica, MA, USA). After blocking with 5% BSA in 1× tris-buffered saline containing 0.1% Tween 20 (pH 7.6) at room temperature for 1 h and incubated with anti-β-catenin (1:1000), anti-Bcl-2 (1:500), anti-Bax (1:500), anti-Cleaved caspase-3 (1:500), anti-E-cadherin (1:500), anti-N-cadherin (1:500), anti-Vimentin (1:1000), anti-Snail (1:500), anti-Claudin-1 (1:500), anti-Slug (1:500), and anti-Twist (1:500) (all from Cell Signaling Technologies, Danvers, MA, USA), and anti-Wnt-5β (1:1000) (Abcam plc, Cambridge, UK), antibodies were stored overnight at 4 °C. Anti-β-actin antibody (1:5000; Sigma-Aldrich, St. Louis, MO, USA) was used as an internal control. Membranes were washed with 1 × Tris-Buffered Saline, 0.1% Tween 20 (TBST) and incubated with horseradish peroxidase-conjugated secondary antibodies (Jackson Immunoresearch, West Grove, PA, USA) for 2 h at room temperature. After washing again with TBST, signal was detected using an enhanced chemiluminescence kit (Thermo Scientific, Rockford, IL, USA), and intensity was quantified using Image J software. The data were obtained from at least three biological replicates.

### 4.10. Flow Cytometry Apoptosis Analysis

Experiments for apoptosis were performed using the Annexin V-FITC Apoptosis Detection Kit (BD Pharmingen, San Diego, CA, USA). The experiment proceeded according to the manufacturer’s instructions. For apoptosis analysis, SKOV3 and OVCA429 cells were transfected with siRNA for 48 h, sorted using FACS Canto II (BD Biosciences, Franklin Lakes, NJ, USA), and analysed with BD FACS Diva software version 6.2. Analysis was performed in triplicate.

### 4.11. Flow Cytometry Cell Cycle Analysis

SKOV3 and OVCA429 cells after siRNA transfection were seeded in 6-well plates and left for attachment overnight at 37 °C. The cells were incubated with siRNA at 37 °C for 48 h before harvesting, washed twice, and resuspended in 0.5 mL of PBS containing 0.2% bovine serum albumin (BSA). The cells were fixed in cold 70% ethanol at 4 °C for at least 1 h. After washing twice with PBS, the cells were resuspended in 0.5 mL of propidium iodide (PI)/RNase staining solution (GeneAll Biotechnology, Seoul, Korea) and incubated at 4 °C for at least 2 h. The cells were analysed with a BD FACSCanto II flow cytometer (BD Bioscience, Franklin Lakes, NJ, USA). The cell cycle distribution was determined using the PI fluorescence signal at FL2-A peak versus count.

### 4.12. Xenograft in Mice

All animal experiment protocols were approved by the Institutional Animal Care and Use Committee of Catholic University. Five-week-old female BALB/c nude mice (*n* = 5, Orient Bio, Seongnam, Korea) were randomized. Each mouse received a subcutaneous injection of 100 μL suspension of OVCA429 cells (1 × 10^7^) that was placed into the dorsal scapular area. Calipers were used to measure tumour size twice per week. Tumour volume was calculated using a simplified equation to estimate a rotational ellipsoid (length × width^2^ × 0.5). When the tumours were approximately 100 mm^3^ in size, mice were then assigned randomly to one of the two groups (*n* = 5 per group). Treatment was started 9 days after transplantation and performed for 3 weeks as follows: Group 1, intratumoural negative control siRNA (30 nM) every twice a week and Group2, siE2F4as (30 nM) every twice a week. The mice were then sacrificed 35 days after the first day of treatment and the tumours were harvested and weighed.

### 4.13. Statistical Analysis

Statistical analysis was performed with SPSS (SPSS Inc., Chicago, IL, USA) and verified through Pearson’s χ^2^ test. Student’s t-test was used for parameter and nonparametric variables. Two assays were used to evaluate the association between the expression of *E2F4as* and clinical pathological characteristics. PFS and ovarian cancer (OC) were analysed by the Kaplan–Meier method, and group differences were estimated using the log-rank test. Multivariate survival analysis of variables of interest was performed using the Cox regression model adjusted for known prognostic covariates (age, stage, tumour grade, lymph node metastasis, and residual tumour). CCK-8 assay, wound healing assy, invasion assay, and qRT-PCR data were all analyzed by t-test, and western blot data were analyzed according to two-way analysis of variance (ANOVA).

## 5. Conclusions

Our research demonstrated that lncRNA *E2F4as* could act as an oncogene in ovarian cancer progress. Early diagnosis using serum through nonsurgical blood collection is possible, and the potential of lncRNA *E2F4as* was confirmed as a biomarker. It can also be a potential treatment target as a prognosis predictor.

## Figures and Tables

**Table 1:** Clinicopathologic factors in benign ovary serum and ovarian cancer serum samples.

		*E2F4as* Expression		
Variables	*n*	Low	High	*p*-Value ^a^
Age (years, mean ± SD)	108	51.06 ± 9.5	53.75 ± 12.8	0.084
Grade				0.235
1	10	5	5	
2	28	8	20	
3	70	17	53	
Stage				0.006 *
I	26	14	12	
II	21	5	16	
III	31	7	24	
IV	30	4	26	
Lymph-node metastasis				0.004 *
Yes	33	3	30	
No	75	27	48	
BMI (Body mass index)				0.393
25>	75	19	56	
25<	33	11	22	
Tumour size				0.201
2_5.9	30	11	19	
≥6	78	19	59	
Recurrence				0.676
Yes	32	8	24	
No	76	22	54	
Menopause				0.401
Yes	68	17	51	
No	40	13	27	
Cell type				0.168
Serous Carcinoma	66	14	52	
Clear cell carcinoma	7	3	4	
Endometrioid	14	6	8	
Mucinous	16	4	12	
Other	5	2	3	

^a^ Chi-square test or Fisher’s exact test were used to calculate *p*-values. * *p* < 0.05.

**Table 2:** Univariate and multivariate analyses of parameters associated with overall survival in 108 patients with ovarian cancer.

	Univariate Analysis		Multivariate Analysis	
Factor	Hazard Ratio (95% CI)	*p*-Value	Hazard Ratio (95% CI)	*p*-Value
*E2F4as* expression	0.575 (0.187–1.766)	0.327	0.218 (0.048–0.996)	0.049 *
Age (years)	1.044 (0.994–1.095)	0.084	0.996 (0.925–10.73)	0.924
Grade	1.961 (0.653–5.886)	0.216	1.310 (0.343–5.002)	0.693
Stage	1.676 (0.969–2.899)	0.055	2.130 (1.005–4.513)	0.048 *
Lymph-node metastasis	4.527 (1.458–14.056)	0.004 **	4.324 (1.392–13.425)	0.011 *
BMI	2.025 (0.675–6.081)	0.199	1.484 (0.444–4.964)	0.521
Tumour size	0.801 (0.246–2.612)	0.712	2.025 (0.471–8.705)	0.343
Recurrence	2.379 (0.798–7.092)	0.109	0.863 (0.232–3.213)	0.826
Menopause	7.368 (0.957–56.723)	0.024 *	6.985 (0.907–53.809)	0.062
Cell type	0.848 (0.531–1.355)	0.487	1.490 (0.444–4.964)	0.146

* *p* < 0.05, ** *p* < 0.01.

## Supplementary Materials

The following are available online at https://www.mdpi.com/2072-6694/12/12/3626/s1. Table S1: Present-study primer sequences of real-time PCR; Table S2: Present-study siRNA primer sequences; Figure S1: Expression of lncRNA *E2F4as* measured by qRT-PCR and MTT assay in SKOV3 and OVCA429 cell lines of siRNA-*E2F4as* transfection; Figure S2: The whole western blot for Figure 4; Figure S3: The whole western blot for Figure 5; Figure S4: EMT-associated genes were analysed by qRT-PCR in mouse tumour tissues for Figure 6.

## Abbreviations

E2F4as	E2F4 antisense
EMT	epithelial-mesenchymal transition
FIGO	The International Federation of Gynecology and Obstetrics
KCLB	Korean Cell Line Bank
siRNA	small interfering RNA
siE2F4as	E2F4as-targeting siRNA
siNC	negative control siRNA
qRT-PCR	quantitative real-time PCR
CCK-8	Cell Counting Kit-8
RIPA	radioimmunoprecipitation assay
HR	hazard ratio
NRF	National Research Foundation of Korea
