# Supplementary material for: Long Noncoding RNA E2F4as Promotes Progression and Predicts Patient Prognosis in Human Ovarian Cancer
Source: Cancers (Basel). 2020 Dec 3;12(12):3626. doi: 10.3390/cancers12123626 (PMC7761684; doi:10.3390/cancers12123626)
Supplement: Supplementary file 1 [file cancers-12-03626-s001.pdf]

cancers

MDPI

Article

Long Noncoding RNA *E2F4as* Promotes Progression and Predicts Patient Prognosis in Human Ovarian Cancer

Sun-Ae Park, Lee Kyung Kim, Young Tae Kim, Tae-Hwe Heo and Hee Jung Kim

Supplementary Materials:

Table S1. Primer sequences used in the present study.

Gene	Primer Sequence		Product Size (bp)
	Forward (5′-3′)	Reverse (5′-3′)	
E2F4as	GTGTCCCTGTCTCCTGTGGT	TGAGCTCACCACTGTCCTTG	228
E-cadherin	CGACCCAACCCAAGAATCTA	AGGCTGTGCCTTCCTACAGA	172
N-cadherin	GACAATGCCCCTCAAGTGTT	CCATTAAGCCGAGTGATGGT	179
β-catenin	TCATGCGTTCTCCTCAGATG	AATCCACTGGTGAACCAAGC	186
Wnt-5β	TCTCAAGAGAGCGAGAAGACT	CTGCACCGGGTTCAAAGCTA	191
Twist	CCGTGGACAGTGATTCCCAG	CCTTTCAGTGGCTGATTGGC	177
Snail	GCGAGCTGCAGGACTCTAAT	CCCACTGTCCTCATCTGAGA	233
Vimentin	TCCTCACATTCGAGCAAAGA	ATTCAAGTCTCAGCGGGCTC	161
Claudin-1	CCTCCTGGGAGTGATAGCAAT	GGCAACTAAAATAGCCAGACCT	145
Slug	CGAACTGGACACACATACAGTG	CTGAGGATCTCTGGTTGTGGT	87
E2F4	AGCGGCGGATTTACGACATT	AATCTCCCGGGTATTGCAGC	116
U6	CTCGCTTCGGCAGCACA	AACGCTTCAGGAATTTGCG	92
GAPDH	TCGACAGTCAGCCGCATCTTCTTT	ACCAAATCCGTTGACTCCGACCTT	186
18s	GATATGCTCATGTGGTGTTG	AATCTTCTTCAGTCGCTCCA	549

Table S2. siRNA primer sequences used in the present study.

No	siRNA name	Duplex sequence	
1	E2F4as-1	Sense	5′ CGGAUUUACGACAUUACCAUU 3′
		Antisense	5′ UGGUAAUGUCGUAAAUCCGUU 3′
2	E2F4as-2	Sense	5′ GAGAUUGCUGACAAACUGAUU 3′
		Antisense	5′ UCAGUUUGUCAGCAAUCUCUU 3′
3	E2F4as-3	Sense	5′ GGACAUCUGCAGAUGCUUUUU 3′
		Antisense	5′ AAAGCAUCUGCAGAUGUCCUU 3′
4	E2F4as-4	Sense	5′ GUCUCAAUGGGCAGAAGAAUU 3′
		Antisense	5′ UUCUUCUGCCCAUUGAGACUU 3′
5	E2F4as-5	Sense	5′ CCAGAUUCACCUGAAGAGUUU 3′
		Antisense	5′ ACUCUUCAGGUGAAUCUGGUU 3′
6	Negative Control	Sense	5′ CCUCGUGCCGUUCCAUCAGGUAGUU 3′
		Antisense	5′ CUACCUGAUGGAACGGCACGAGGUU 3′

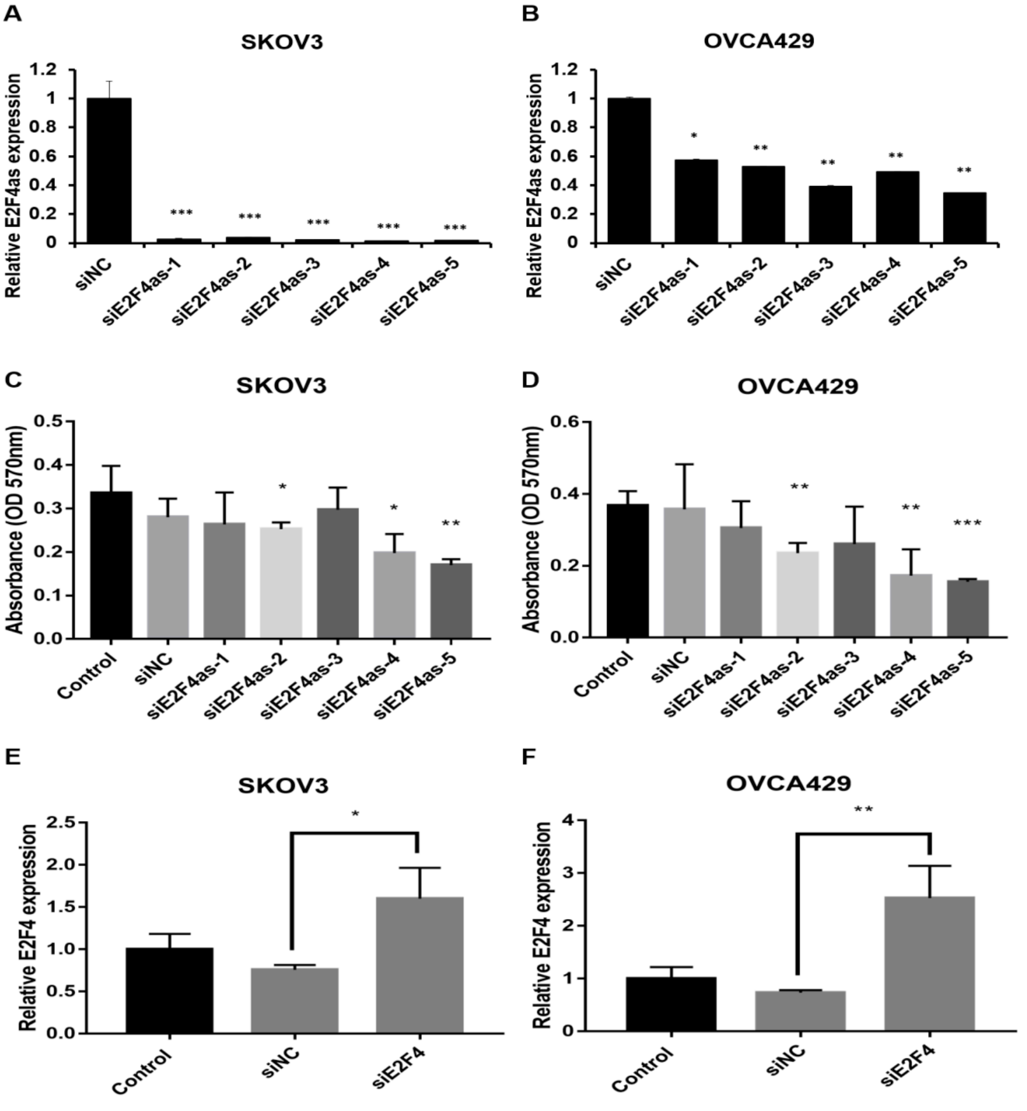

Figure S1. The experiment to select siE2F4as in SKOV3 and OVCA429 cell lines. (A, B) The expression of lncRNA *E2F4as* was measured by qRT-PCR in SKOV3 and OVCA429 cell lines of siRNA-*E2F4as* transfection. siE2F4as selected siE2F4as-5, which had the most decreased expression of *E2F4as* through qRT-PCR among several siRNAs. (C, D) siE2F4as selected siE2F4as-5, which had the most decreased expression of *E2F4as* through and MTT assay among several siRNAs. Data expressed as mean ± standard deviation; *p < 0.05, **p < 0.01, ***p < 0.001 vs. siNC. (E, F) The expression of E2F4 was measured by qRT-PCR in SKOV3 and OVCA429 cell lines of si-RNA-E2F4as transfection. Data expressed as mean ± standard deviation; *p < 0.05, **p < 0.01 vs siNC.

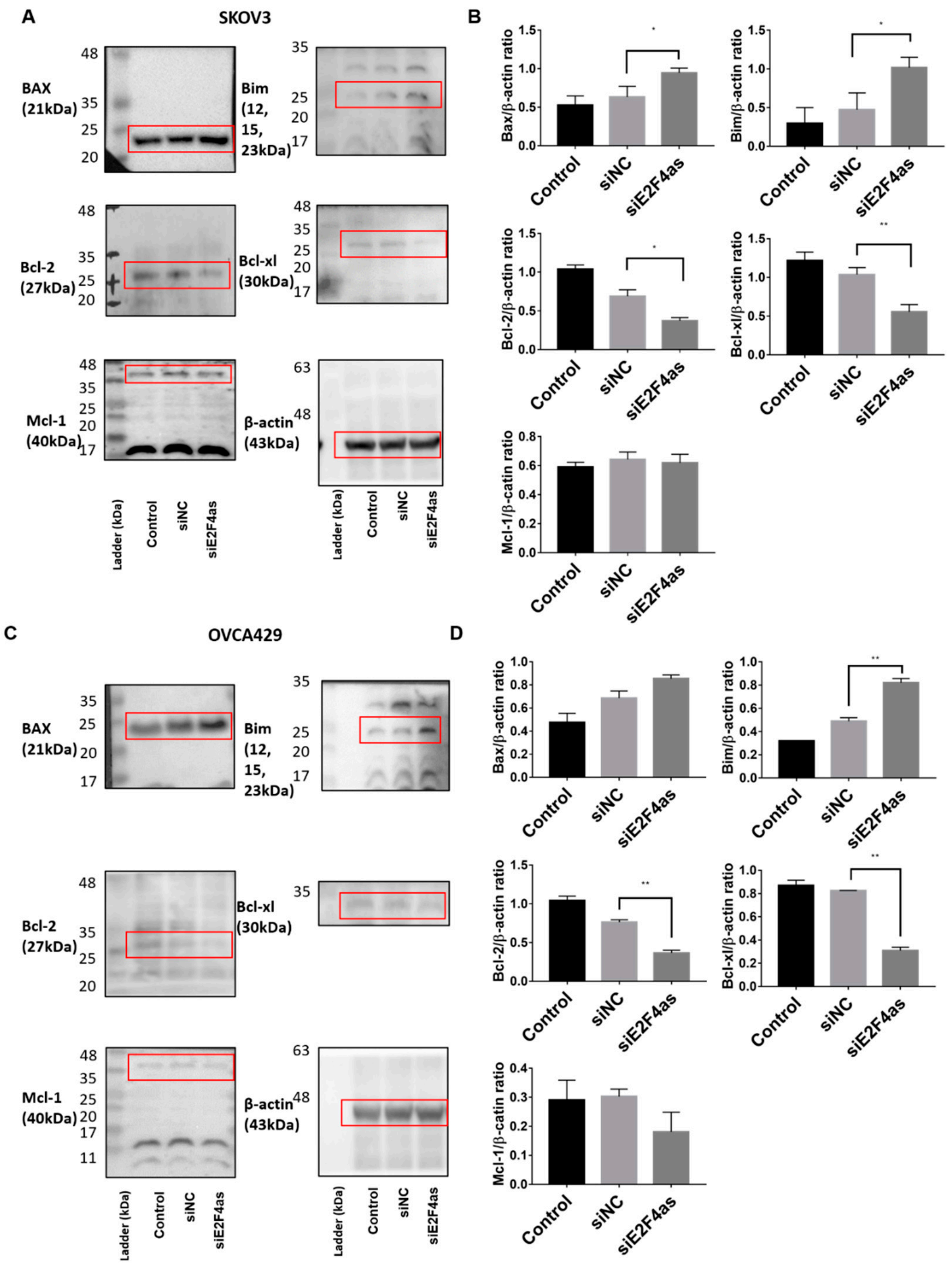

Figure S2. The Whole Western Blot for Figure 4. (A, C) Protein lysates obtained from SKOV3 and OVCA429 cells 48 h after transfection with *E2F4as* siRNA (30nM) or negative control. Levels of proteins in pro-apoptosis and anti-apoptosis-associated genes analysed using Western blotting. (B, D) Data were presented as mean ± SD from three independent experiments (*$p < 0.05$, **$p < 0.01$, identified by two-way ANOVA vs siNC).

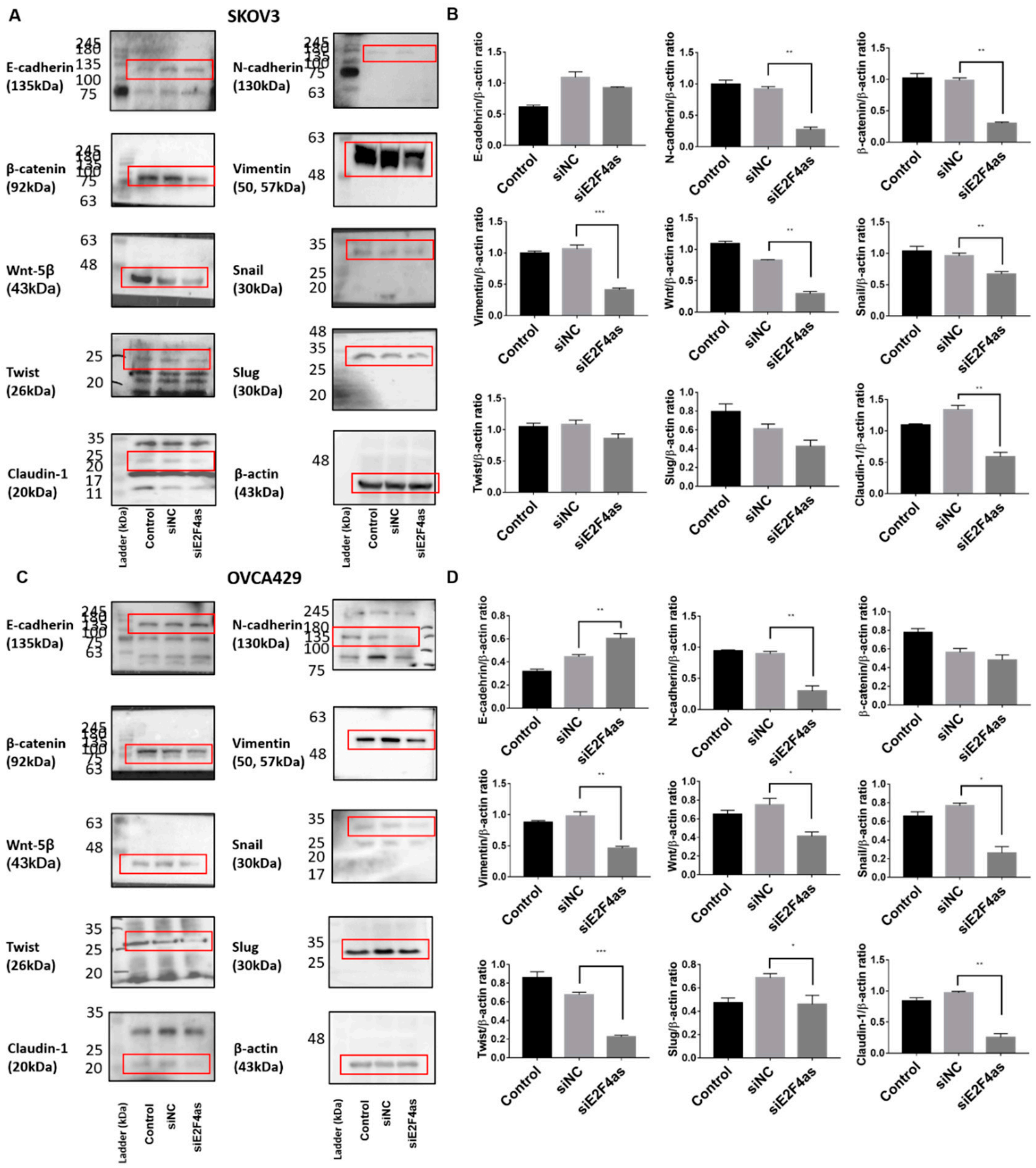

Figure S3. The Whole Western Blot for Figure 5. (A, C) Protein lysates obtained from SKOV3 and OVCA429 cells 48 h after transfection with *E2F4as* siRNA (30nM) or negative control. Levels of E-cadherin, N-cadherin, β-catenin, Wnt-5β, Twist, Snail, Slug, Vimentin, and Claudin-1 analysed by Western blotting. (B, D) Data were presented as mean ± SD from three independent experiments (*$p < 0.05$, **$p < 0.01$, ***$p < 0.001$, identified by two-way ANOVA vs siNC).

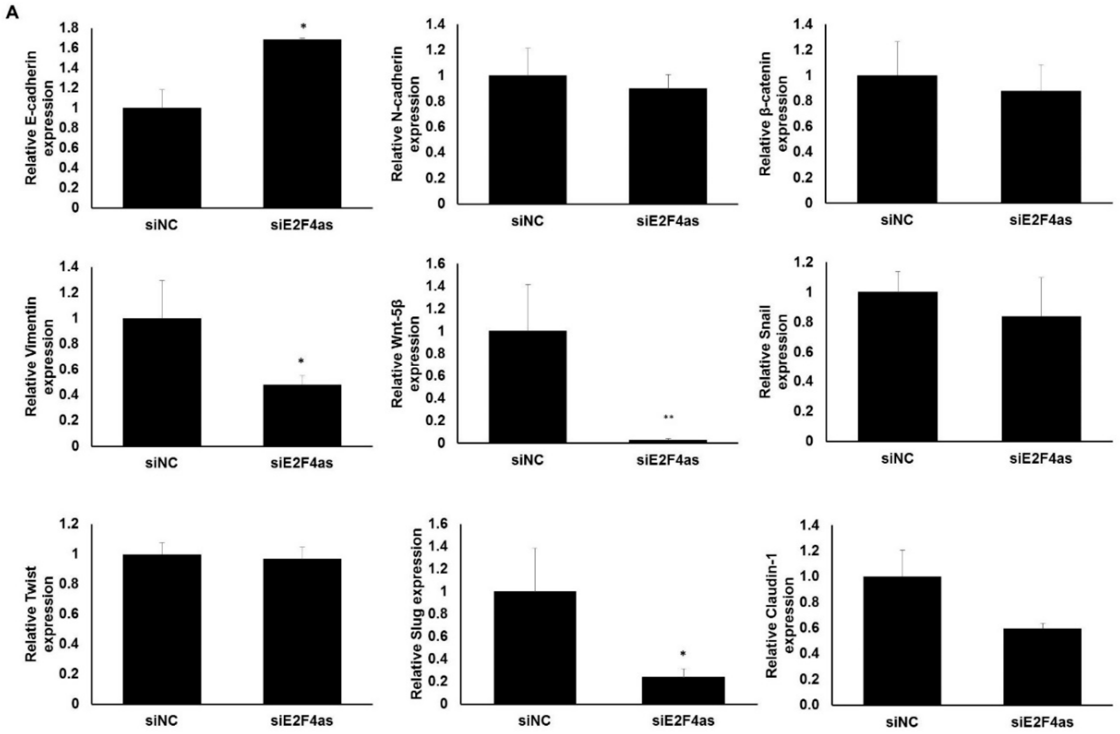

Figure S4. EMT-associated genes were analysed by qRT-PCR in mouse tumour tissues. (**A**) Levels of E-cadherin, N-cadherin, β-catenin, Wnt-5β, Twist, snail, slug, Vimentin and Claudin-1 were analyzed by qRT-PCR in mouse tumour tissues. Data expressed as mean ± standard deviation; **p* < 0.05, ***p* < 0.01 vs siNC.

© 2020 by the authors. Submitted for possible open access publication under the terms and conditions of the Creative Commons Attribution (CC BY) license (http://creativecommons.org/licenses/by/4.0/).
